# YKL-40, Soluble IL-2 Receptor, Angiotensin Converting Enzyme and C-Reactive Protein: Comparison of Markers of Sarcoidosis Activity

**DOI:** 10.3390/biom8030084

**Published:** 2018-08-28

**Authors:** Pelin Uysal, Sinem Durmus, Volkan Sozer, Remise Gelisgen, Ekrem Cengiz Seyhan, Fusun Erdenen, Gonul Simsek, Hafize Uzun

**Affiliations:** 1 Department of Chest Diseases, Medical Faculty, Acıbadem University, 34752 Istanbul, Turkey; drpelinuysal@gmail.com; 2 Department of Biochemistry, Cerrahpasa Medical Faculty, Istanbul University, 34098 Istanbul, Turkey; durmus.sinem@gmail.com (S.D.); remisagelisgen@hotmail.com (R.G.); 3 Department of Biochemistry, Yildiz Technical University, 34220 Istanbul, Turkey; vsozer@gmail.com; 4 Department of Chest Diseases, Medical Faculty, Medipol University, 34810 Istanbul, Turkey; drekremcs@yahoo.com; 5 Istanbul Training and Research Hospital, Department of Internal Medicine, 34098 Istanbul, Turkey; fusunozerdenen@hotmail.com; 6 Department of Physiology, Cerrahpasa Medical Faculty, Istanbul University, 34098 Istanbul, Turkey; gdincsimsek@yahoo.com

**Keywords:** sarcoidosis, chitinase-3-like protein 1 (YKL-40), soluble interleukin-2 receptor (sIL-2R), high-sensitive C-reactive protein (hs-CRP), adenosine deaminase (ADA), angiotensin converting enzyme (ACE), neopterin (NEO)

## Abstract

The aims of this study were to describe the clinical, radiological and immunological features of a population of sarcoidosis patients and to analyse chitinase-3-like protein 1 (YKL-40), soluble interleukin-2 receptor (sIL-2R), neopterin concentrations and adenosine deaminase (ADA) activity in serum of these patients in order to understand their potential as disease markers. Fifty-nine patients affected by chronic sarcoidosis, in active (20 patients) and inactive (39 patients) phase according to the clinical, radiological and laboratory criteria were studied. Serum YKL-40, sIL-2R, high-sensitive C-reactive protein (hs-CRP), neopterin levels and ADA activities were evaluated and compared with those of 25 healthy controls. Individuals with chronic sarcoidosis were significantly higher serum YKL-40, sIL-2R, neopterin, hs-CRP concentrations, angiotensin converting enzyme (ACE) and ADA activity than those of control subjects. Sarcoidosis patients in the active phase of the disease were significantly higher YKL-40, sIL-2R, hs-CRP levels and ACE activity than those in the inactive phase, while ADA activities and neopterin levels did not display any significant difference between the active and inactive disease groups. In comparison to the other parameters, as panel measurement of the serum YKL-40, sIL-2R, ACE and hs-CRP indicate a greater discrimination between active and inactive disease. The results indicate that serum YKL-40, sIL-2R, ACE and hs-CRP concentrations may be useful marker for monitoring sarcoidosis disease activity.

## 1. Introduction

Sarcoidosis is a chronic, progressive, systemic disease of an unknown aetiology, characterized by the formation of noncaseating granulomas in the affected organs. It may involve the lungs, skin, eyes, and the myocardial, osteoarticular, reticuloendothelial and central nervous systems [1]. Angiotensin converting enzyme (ACE) and soluble interleukin-2 receptor (sIL-2R) are the most widely used serum markers of sarcoidosis; they can easily be detected in the serum and bronchoalveolar lavage (BAL) fluid of sarcoidosis patients and have relative prognostic value. Soluble interleukin-2 receptor (sIL-2R) is a T-cell surface receptor, elevated levels of which have been described in serum and BAL fluid of sarcoidosis patients related to the increased number and enhanced activation of T-lymphocytes characteristic of this disease. Soluble interleukin-2 receptor (sIL-2R) has been shown to be an accurate parameter in the assessment of sarcoidosis and correlates with active disease, although it is still not recommended as an activity marker [2].

Adenosine deaminase (ADA) is an enzyme involved in the purine catabolism, produced by mononuclear cells and lymphocytes. Serum ADA activity has been shown to be increased in sarcoidosis, especially in untreated patients and it has been suggested that ADA assay may be useful as a marker of the activity of the disease [3]. C-reactive protein (CRP) is an acute-phase reactant with well-documented sensitivity that is commonly used to diagnose infectious and inflammatory conditions. The CRP response (although rather minor) was present in the majority of patients and appeared indicative of the inflammatory activity of sarcoidosis [4]. High-sensitivity C-reactive protein (hs-CRP) methods have recently been introduced to accurately monitor minor increases in serum CRP but limited studies evaluating hs-CRP in sarcoidosis have been reported [5]. The human cartilage glycoprotein-39, or YKL-40 (its name derived from the first three N-terminal amino acids and from its molecular weight of 40 kDa) has recently shown its potential merit as a novel marker for sarcoidosis [6]. However, little is known concerning serum levels of YKL-40 in sarcoidosis patients.

Many attempts have been made to find serological biomarkers of disease activity in sarcoidosis, which could help identify patients at risk for irreversible organ damage, for example, lung fibrosis [7]. However, there is still no single specific marker with unequivocal prognostic significance.

Clinical, radiological and physiologic parameters have been used to determine the activity and severity of the disease nowadays and a search for a compatible serum marker is also on the front burner. The diagnosis of sarcoidosis can be challenging even for experienced patients, because there is currently no single diagnostic test for it. Despite the fact that biopsy is not required in some cases, diagnosis of sarcoidosis is often confirmed by invasive tissue biopsy for the majority of patients. For this reason, it is clear that there is a great need for the development of noninvasive diagnostic methods. Although some new biomarkers have been discovered in recent years, there are no gold standard laboratory tests to facilitate early recognition.

The aims of this study were to describe the clinical, radiological and immunological features of a population of chronic sarcoidosis patients and to analyse YKL-40, sIL-2R, ACE, hs-CRP, neopterin concentrations and ADA activities in serum of these patients in order to understand their potential as disease markers. We aimed to investigate the diagnostic features of YKL-40, sIL-2R, ACE, hs-CRP, neopterin concentrations and ADA markers in active and inactive discrimination of sarcoidosis disease.

## 2. Materials and Methods

The protocol was approved by the local Ethics Committee of Istanbul Education and Research Hospital and was conducted in accordance with the Declaration of Helsinki. All participants were informed about the study and they signed informed consent.

Twenty active sarcoidosis subjects (male/female (M/F): 4/16) aged 45.15 ± 11.55 years-old, 39 inactive subjects (M/F: 15/24) aged 42.4 ± 10.4 years-old with chronic sarcoidosis and 25 control subjects (M/F: 12/13) aged 46.1 ± 8.4 years-old were recruited in our study. The activation of the disease was determined according to WASOG (Consensus of World Association of Sarcoidosis and other Granulomatous Diseases-1999) criteria (symptoms, X-ray findings, lung diffusion capacity for carbon monoxide (DLCO), liver functions, ball findings) [8]. Definition of active and inactive disease: (1) Clinically; (i) the presence of eritema nodosum; (ii) arthritis; (iii) exercise dyspnoea; (iv) new-onset extrapulmonary organ involvement; (2) In terms of pulmonary functions; (i) at least 15% difference between forced expiratory volume in one second (FEV1) and forced vital capacity (FVC) values in pulmonary function tests performed with three-month intervals; (ii) at least 10% difference between DLCO values in pulmonary function tests performed with three-month intervals; (3) Radiologically; (i) the emergence of a new lymph node; (ii) existing lymph node doubling or decreasing the volume; (iii) a newly developed diffuse interstitial and/or alveolar type radiological pattern. If at least one of the above is present, the patient is evaluated as active [9,10,11,12,13,14]. Patients were selected from our multidisciplinary referral setting (RS) at Istanbul Education and Research Hospital, İstanbul, Turkey. This referral setting has been acting to diagnose or to confirm the diagnose firstly, search for the extrapulmonary manifestations, treat and follow up these patients. The diagnosis and extent of the disease were determined based on typical clinical, radiological and laboratory criteria, together with the presence of noncaseating granulomas in biopsy specimens. The referrals to our centre came either from the various departments of our faculty or from outside physicians. All patients are initially evaluated by an internalist. The records were reviewed initially to confirm the diagnosis of sarcoidosis. The medical records and when indicated, the radiographic findings of each person with a diagnosis of sarcoidosis were reviewed by a senior author. Patients who were suspected of having sarcoidosis underwent a biopsy of the most accessible involved organ. All patients were free of vascular and renal diseases (serum creatinine < 1.3 mg/dL), malignancy, connective tissue diseases, endocrine diseases and alcoholism.

Case Definitions:

- In treatment: Patients who started corticosteroid therapy after diagnosis.
- Spontaneous Remission: Spontaneous clinical and/or radiological improvement.
- Treatment-related remission: Clinical/radiological improvement following cessation of corticosteroid therapy.
- Chronic disease: if the disease is not more than two years.

The controls that were taken into operation were selected from those who came to the check-up polyclinic and had no disease.

### 2.1. Sample Collection

Fresh blood samples were drawn after 12–14 h of fasting in the morning. Serum was obtained after at least 30 min of clotting by centrifugation at 2500× *g* for 15 min. Serum was stored at −80 °C until assayed for determination of all parameters. All icteric or haemolytic blood samples were discarded.

### 2.2. Assay of Biochemical Parameters

The ADA activity was analysed using a commercial colorimetric assay kit (Diazyme General Atomics, Poway, CA, USA). Intra and inter-coefficients of variances (CV) for ADA activity were 6.6% and 8.4%, respectively.

The serum neopterin levels were measured by a solid-phase competitive enzyme-linked immunosorbent assay (IBL, Hamburg, Germany). Intra and inter-CV for neopterin levels were 5.3% and 7.2%, respectively.

The serum YKL-40 levels were assayed by a sandwich immunoassay in a microtitre stripwell format (Chondrex, Metra Biosystems Inc., Mountainview, CA, USA). Intra and inter-CV for YKL-40 levels were 5.5% and 7.0%, respectively.

The serum sIL-2R levels were determined by enzyme-linked immunoassay (Bender Med Systems, Vienna, Austria). Intra and inter-CV for sIL-2R levels were 4.7% and 6.3%, respectively.

The serum hs-CRP levels were measured by particle enhanced immunonephelometry on the BN Prospec (Dade Behring, Deerfield, IL, USA). Intra and inter-CV for hs-CRP levels were 3.9% and 5.7%, respectively.

The serum ACE levels were determined by enzyme-linked immunoassay (Quantikine, R&D Systems, Minneapolis, MN, USA). Intra and inter-CV for ACE levels were 4.3% and 5.9%, respectively.

### 2.3. Statistical Analysis

Statistical analysis was performed by using the statistical package for the social sciences (SPSS) (version 21.0, SPSS Inc., Chicago, IL, USA). All data were first checked for normality. Categorical variables were presented as absolute numbers and ratio and analysed using either the Chi-square test (*χ*^2^) or Fisher’s exact test. Normally distributed continuous variables were presented as mean ± standard deviation (SD) and analysed by one-way ANOVA (analysis of variance) followed by Tukey multiple comparison tests. Differences among two groups were analysed with Student’s *t*-test. Pearson’s correlations were used to test the relationship among variables. The area under the receiver operating characteristic (ROC) curve, known as the AUC curve analyses were used to compare the diagnostic accuracy of different markers to assess disease activity. Significant variables were included into multivariate analysis. Differences were considered significant when *p* <  0.05. Power analysis was used to perform calculations on sample size, effect size and statistical power. The minimal significance (*α*) and statistical power (1 − *β*) were set at 0.05 and 0.80 respectively.

## 3. Results

**Figure 1:**
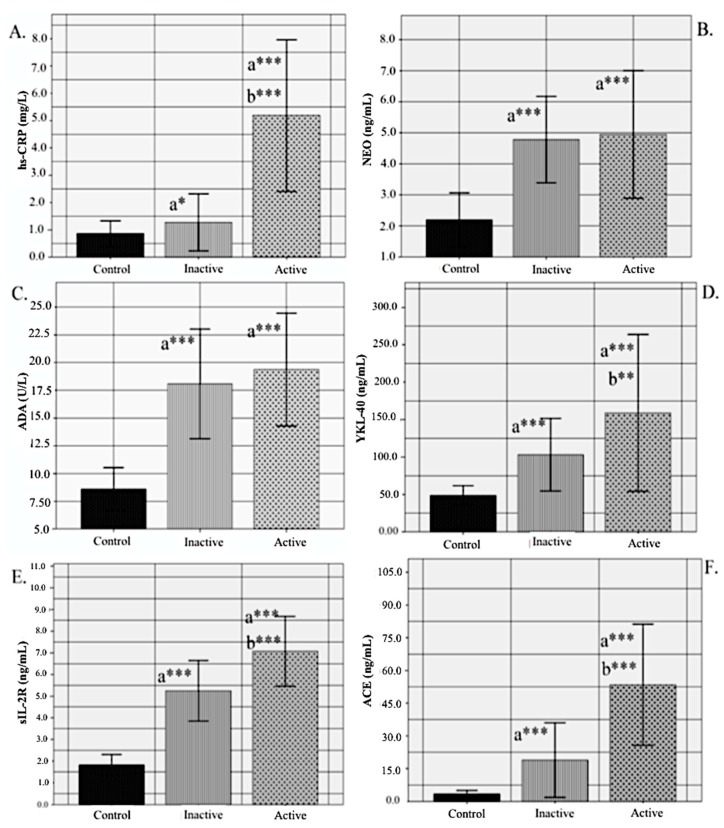
Biochemical parameters in control, active and inactive sarcoidosis patients, (**A**) High-sensitivity C-reactive protein (hs-CRP) (mg/L); (**B**) Neopterin (NEO) (ng/mL); (**C**) Adenosine deaminase (ADA) (U/L); (**D**) Chitinase-3-Like Protein 1 (YKL–40) (ng/mL); (**E**) Soluble interleukin-2 receptor (sIL-2R) (ng/mL); (**F**) Angiotensin-converting enzyme ACE (ng/mL). a: vs. control; b: vs. inactive sarcoidosis. * *p* < 0.05; ** *p* < 0.01; *** *p* ≤ 0.001.

Subject characteristics and circulating concentrations of biochemical markers are given in Table 1. Both active and inactive sarcoidosis patients had statistical significantly higher neopterin, ADA, YKL-40, sIL-2R and ACE levels (for all *p* < 0.001) than control subjects; and also, the hs-CRP levels of both patient group were greater than controls (for active sarcoidosis patients *p* < 0.001; for inactive sarcoidosis patients *p* < 0.05). Also, hs-CRP (*p* < 0.001), YKL-40 (*p* < 0.01), sIL-2R (*p* < 0.001) and ACE (*p* < 0.001) levels were elevated in active sarcoidosis patients compared to inactive patients (Figure 1). 

**Figure 2:**
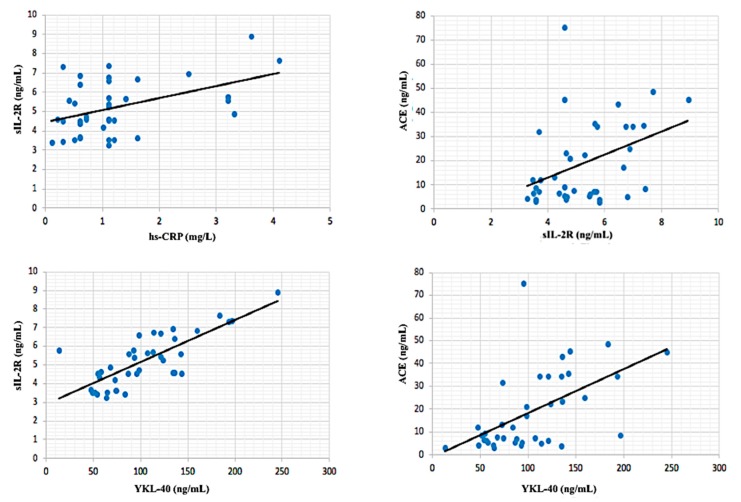
Correlations between biochemical parameters in patients with inactive sarcoidosis.

**Figure 3:**
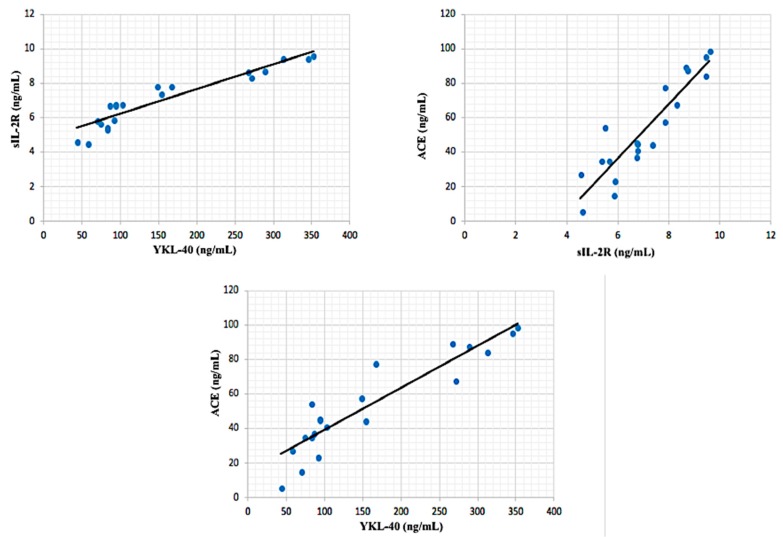
Correlations between biochemical parameters in patients with active sarcoidosis.

**Figure 4:**
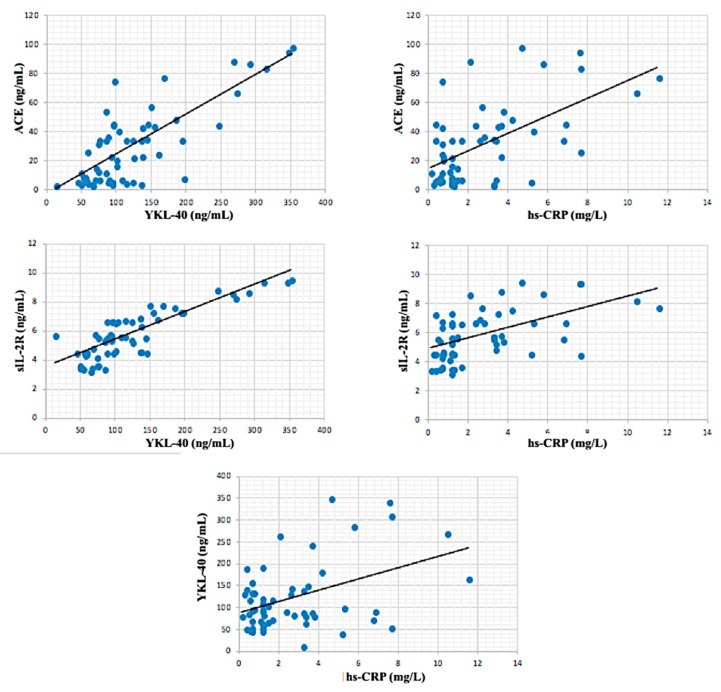
Correlations between biochemical parameters in all patients (active + inactive sarcoidosis).

In inactive sarcoidosis patient group, hs-CRP was moderately correlated with age (*r*: correlation coefficient) (*r* = 0.427; *p* < 0.01) and sIL-2R (*r* = 0.464, *p* < 0.01). Also, ACE was moderately correlated with sIL-2R (*r* = 0.40, *p* < 0.05) and ACE (*r* = 0.546, *p* < 0.001). There was a significant strong positive correlation between sIL-2R and YKL-40 (*r* = 0.779, *p* < 0.001) (Figure 2). In active sarcoidosis group, the closest correlation was observed between sIL-2R and YKL-40 (*r* = 0.939, *p* < 0.001). Also, ACE was very strongly correlated with YKL-40 (*r* = 0.919, *p* < 0.001) and ACE (*r* = 0.914, *p* < 0.001) (Figure 3). A significant very strong correlation between sIL-2R and YKL-40 was found in all patient groups (active + inactive sarcoidosis). Additionally, ACE was strongly correlated with YKL-40 (*r* = 0.788, *p* < 0.001) and sIL-2R (*r* = 0.788, *p* < 0.001). However, hs-CRP was moderately correlated with YKL-40 (*r* = 0.432, *p* < 0.001), sIL-2R (*r* = 0.547, *p* < 0.001) and ACE (*r* = 0.584, *p* < 0.001) in all patient group (Figure 4).

**Figure 5:**
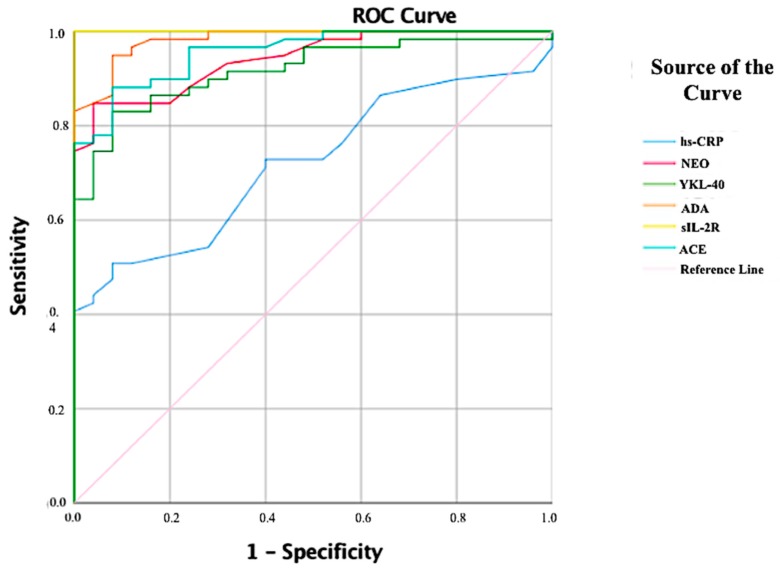
Receiver operating characteristic (ROC) analysis for biochemical parameters of all patients (active + inactive sarcoidosis).

**Figure 6:**
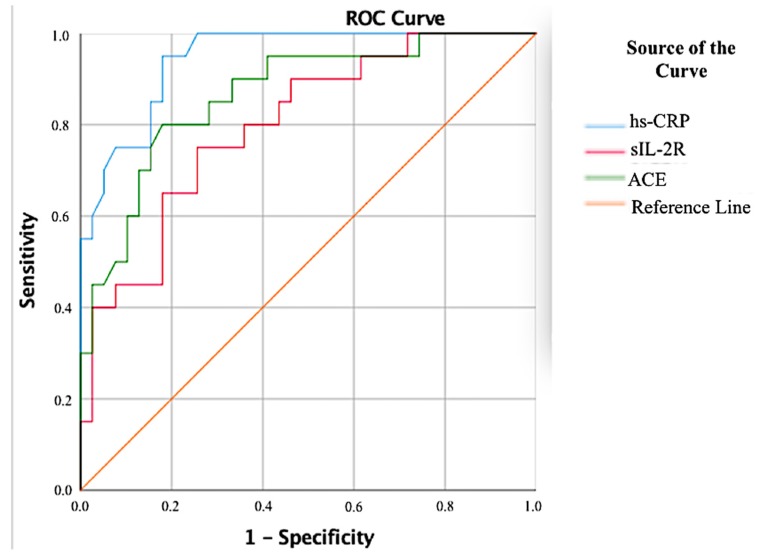
ROC analysis for biochemical parameters of patients with active sarcoidosis.

The effectiveness of the investigated biochemical parameters in the early recognition of patients with active and inactive sarcoidosis was assessed using ROC analysis. Figure 5 shows the ROC curves for all biochemical parameters that we investigated of all of the patients. The sIL-2R levels had very good diagnostic value in differentiating between the control and patients ((AUC) = 1.000, *p* < 0.001, 95% confidence interval (CI): 1.00–1.00). Additionally, the AUC of ADA was 0.983 (95% CI 0.962–1.00), of ACE was 0.956 (95% CI 0.918–0.994), of neopterin was 0.942 (95% CI 0.897–0.987), of YKL-40 was 0.917 (95% CI 0.859–0.976), whereas the AUC of hs-CRP was 0.721 (95% CI 0.614–0.828) (Table 2). Figure 6 also shows the all ROC curve for patients with active sarcoidosis and there was no significant difference in diagnostic values of neopterin ((AUC) = 0.501, 95% CI 0.327–0.675), YKL-40 ((AUC) = 0.630, 95% CI 0.471–0.790) and ADA ((AUC) = 0.562, 95% CI 0.405–0.718) between the active and inactive sarcoidosis. Interestingly hs-CRP had the best diagnostic value in differentiating active and inactive sarcoidosis ((AUC) = 0.946, 95% CI 0.894–0.997, sensitivity = 0.95 and specificity = 0.821 for cut off 1.8 mg/L) and the area under the curve for hs-CRP was significantly higher than that for sIL-2R ((AUC) = 0.799, 95% CI 0.682–0.916 and ACE ((AUC) = 0.865, 95% CI 0.767–0.963) (Table 3).

Multivariate analysis revealed an association between the presence of active sarcoidosis and the levels of hs-CRP (OR = 5.162, *p* = 0.002) and ACE (OR = 1.085, *p* = 0.029) (Table 4). We also performed a separate multivariate analysis for the cut-off points that we obtained from the ROC analysis, to better identify the separation power of this parameters for active patients. Our second multivariate analysis showed that if hs-CRP is greater than 2 mg/L, the risk of disease activity increases 47.225-fold (*p* = 0.001) (Table 5).

## 4. Discussion

Activity in sarcoidosis is the continuation of clinical radiological and physiological changes. The activity of the disease in this disease, which involves many systems, is important in determining the sequelae and duration of this treatment, treatment decision and clinical follow-up. This study showed that YKL-40, sIL-2R, ACE and hs-CRP were elevated in active sarcoidosis patients compared to inactive patients. When the results of our study are taken into consideration, we also believe that YKL-40, sIL-2R, ACE and hs-CRP as activity panel indicator will be useful.

Currently, clinical, radiological and physiological parameters are used to determine the activity and severity of the disease and a search for serologic searchers compatible with them is also on the agenda. In our study, we determined the activity of the disease by clinical, radiological and physiological parameters. Sarcoidosis is characterized by an excessive immune response to an unknown antigen [15]. Markers of a disease such as sarcoidosis should serve a variety of purposes. As a diagnostic test, determine whether the disease is present, determine whether the disease is active as an activity marker and predict the disease progression as the prognostic factor, the long-term outcome of the disease. Most cytokines as interleukins (IL) sIL-2, IL-5, IL-7, IL-17, IL-18 and granulocyte-macrophage colony-stimulating factor (GM-CSF) and activated cells are only found in the involved organs and not in the peripheral blood [15]. Serum sIL-2R levels are a marker for T-cell activations and sIL-2R is also known to be a marker for extrapulmonary involvement [9,16]. However, the results are conflicting [5,7,9,15,17,18,19,20,21,22,23,24,25,26,27,28]. Su et al. [26] found that serum sIL-2R levels did not correlate with pulmonary function measures or severity score. Our study showed that among activity markers, sIL-2R levels were increased in sarcoidosis, so it was promising marker for monitoring sarcoidosis disease activity. Grutters et al. [9] suggested that a role for serum sIL-2R as marker of pulmonary disease activity and extrapulmonary disease in patients with sarcoidosis. They were no found relation between sIL-2R level and response to treatment and there was no association between sIL-2R levels and radiographic evolution and lung function outcome. Further, the negative association between sIL-2R level and blood lymphocytes and the positive association between sIL-2R level and BAL lymphocytes, suggests that serum sIL-2R is not manufactured in the blood but more likely at sites of disease activity (e.g., the alveolar and interstitial spaces) and then released into the blood. Based on this finding, if a high sIL-2R level is detected and if there is no lymphocytosis in the BAL, this should be taken into account and extrapulmonary sarcoidosis should be considered in the patient. They explained this by the fact that IL-2R is produced when the disease is active and then released to blood circulation. Gungor et al. [15] that demonstrated that among the serum markers that were analysed, sIL-2R levels in cases with extra-pulmonary organ involvement were significantly higher than the cases with pulmonary involvement alone. The highest level was seen in a case with two organ involvements (parotid, skin). Rothkrantz-Kos et al. [5] have assessed the levels of CRP, serum amyloid-A protein (SAA), sIL2R and ACE and observed that the pulmonary function impairment was best reflected by the sIL-2R levels. The sIL-2R levels had very good diagnostic value in differentiating between the control and patients in current study. When the results of our study are taken into consideration, we believe that sIL-2R level measurement will be useful in investigating extrapulmonary organ involvement as an activity indicator. Its value as a disease marker of our result in sarcoidosis was confirmed by other study [5,7,9,15,23,24,25].

The best-known marker is ACE activity in sarcoidosis that ACE activities were elevated in active sarcoidosis patients compared to inactive patients in current study. Angiotensin converting enzyme activity was also moderately correlated with sIL-2R in our study. Furthermore, the ACE levels had very good diagnostic value in patients with active sarcoidosis (sensitivity: 0.85; specificity: 0.718). There has been controversy concerning the role of serum ACE as a diagnostic and activity marker for sarcoidosis [11,15,21,22,29,30,31,32,33]. While there are several studies demonstrating that serum ACE levels are elevated in active sarcoidosis, the serum ACE levels were not significantly different between active and inactive sarcoidosis [11,15,29]. Tanimura et al. [29] showed that clinical disease activity has been correlated with serum ACE, lysozyme and sIL-2R in patients with sarcoidosis. Clinical disease activity has been correlated with serum ACE, lysozyme and sIL-2R in patients with sarcoidosis. Their study showed that sCD163 serum levels positively correlated with those of ACE and sIL-2R and significantly decreased after treatments were administered. Angiotensin converting enzyme levels were also shown to be insignificant in determining the severity of the disease and inappropriate to be used during follow-up [5]. Gungor et al. [15] reported that the sensitivity and specificity of serum ACE levels were 72% and 60%, respectively; the serum ACE levels were not significantly different between the cases considered to be active and inactive. Rust et al. [11] found that the radiologic stage and the initial serum ACE could not discriminate between the two groups. Serum ACE had a very high nonpredictive value for sarcoid uveitis, eliminating the need for further screening tests in subjects with normal serum ACE, unless clinical suspicion was high [30,31,32,34,35]. Sarcoidosis is a systemic disease characterized by noncasefied granulomas in various organs. Serum ACE was also higher in patients with diffuse splenic involvement in sarcoidosis [33]. Thus, measuring serum levels of ACE may be helpful for monitoring the disease activity of sarcoidosis.

Chitinase-3-like protein 1 is an inflammatory glycoprotein, a growth factor for fibroblasts and vascular endothelial cells, is secreted by macrophages and neutrophils [36]. In current study, YKL-40 levels were elevated in active sarcoidosis patients compared to inactive patients. Angiotensin converting enzyme was strongly correlated with YKL-40 and sIL-2R. A significant very strong correlation between sIL-2R and YKL-40 was found in all patient groups (active + inactive sarcoidosis). Additionally, ACE was strongly correlated with YKL-40. In this study serum YKL-40 levels were significantly increased in patients with sarcoidosis reflecting its significant role in the pathogenesis of the disease. Chitinase-3-like protein 1 has been proposed as a diagnostic and prognostic biomarker for various forms of interstitial lung disease, especially sarcoidosis [6,37,38,39,40]. Study of Johansen et al. [6] is the first study of YKL-40 in patients with sarcoidosis that serum YKL-40 may be a novel biomarker of sarcoid disease activity and ongoing fibrosis in patients with pulmonary sarcoidosis. Kruit et al. [37] demonstrated that YKL-40 may be used as a sarcoidosis disease marker but it is unsuitable as a marker to predict the course of the disease. A variant (-329 G/A polymorphism) in the chitinase 3 like 1 gene (*CHI3L1*) contributes to interindividual variations of YKL-40 levels but does not influence sarcoidosis disease susceptibility or severity. We propose that the serum YKL-40 level might add to the predictive value for increased mononuclear cells/macrophages in pulmonary sarcoidosis.

The results of our two multivariate analyses also show that hs-CRP remains the most powerful parameter for distinguishing disease activity. Especially, when hs-CRP values are above 2 mg/L, the risk of disease activity increases by nearly 47-fold, which is a significant finding. Nevertheless, we believe that other parameters and especially ACE should be studied in larger populations to assess the risk of disease activity and that promising results may be achieved.

Patients with chronic sarcoidosis were significantly higher serum YKL-40, sIL-2R, neopterin, hs-CRP, ACE and ADA activity than those of control subjects. Sarcoidosis patients in the active phase of the disease were significantly higher YKL-40, sIL-2R, ACE and hs-CRP levels than those in the inactive phase, while ADA activities and neopterin levels did not display any significant difference between the active and inactive disease groups. There was also a positive correlation between the YKL-40 and sIL-2R values in both active and inactive groups with chronic sarcoidosis. In comparison to the other parameters, measurement of the serum YKL-40 sIL-2R, ACE and hs-CRP indicate a greater discrimination between active and inactive disease. In conclusion, our results indicate that serum YKL-40, sIL-2R, ACE and hs-CRP concentrations may be useful panel marker for monitoring sarcoidosis disease activity. Large cohorts and at-risk populations are needed to confirm the predictive value of these findings.

## Figures and Tables

**Table 1:** Demographic data and biochemical parameters in control, active and inactive sarcoidosis patients.

Variables	Control (*n* = 25)	Inactive Sarcoidosis (*n* = 39)	Active Sarcoidosis (*n* = 20)
**Age, mean ± SD**	46.12 ± 8.27	42.44 ± 10.25	45.15 ± 11.26
**Sex (M/F)**	12/13	15/24	4/16 ^a,^*
**Smoking (Y/N)**	0/20	7/32 ^a,^*	5/15 ^a,^**
**BMI**	26 ± 6	27 ± 5	25 ± 4
**Duration of disease**	-	7 (2–3)	6 (4–22)
**Disease stage (I/II/III/IV)**	-	8/20/8/3	4/12/3/1 *
**Lymphocyte count (/mm^3^)**	-	1308 ± 406	1608 ± 106 *

SD: standard deviation; M/F: male/female; Y/N: yes/no; BMI: body mass index; ^a^: vs. control. * *p* < 0.05; ** *p* < 0.01.

**Table 2:** ROC analysis for biochemical parameters of all patients (active + inactive sarcoidosis). Bold printed are the indicators with statistically significant difference.

	AUC	95% Confidence Interval	*p*	Sensitivity	Specificity	Cut-off
**hs-CRP (mg/L)**	0.721	0.614–0.828	0.001	0.508	0.92	1.35
**NEO (ng/mL)**	0.942	0.897–0.987	0.000	0.881	0.76	2.85
**YKL-40 (ng/mL)**	0.917	0.859–0.976	0.000	0.831	0.920	62.88
**ADA (U/L)**	0.983	0.962–1.00	0.000	0.949	0.92	11.35
**sIL-2R (ng/mL)**	1.000	1.00–1.00	0.000	1.00	1.00	2.97
**ACE (ng/mL)**	0.956	0.918–0.994	0.000	0.881	0.92	5.37

AUC: Area under the curve, hs-CRP**:** high-sensitivity C-reactive protein, NEO: neopterin, ADA: adenosine deaminase, YKL-40, chitinase-3-like protein 1, sIL2R: soluble interleukin-2 receptor, ACE: Angiotensin converting enzyme.

**Table 3:** ROC analysis for biochemical parameters of patients with active sarcoidosis.

	AUC	95% Confidence Interval	*p*	Sensitivity	Specificity	Cut-off
**hs-CRP (mg/L)**	0.946	0.894–0.997	0.000	0.950	0.821	1.8
**sIL-2R (ng/mL)**	0.799	0.682–0.916	0.000	0.750	0.744	5.810
**ACE (ng/mL)**	0.865	0.767–0.963	0.000	0.85	0.718	26.23

**Table 4:** Multivariate logistic regression analysis of biochemical parameters for biochemical parameters.

	OR	*p*
**hs-CRP (mg/L)**	**5.162**	**0.002**
**sIL-2R (ng/mL)**	0.786	0.617
**ACE (ng/mL)**	1.085	0.029

Bold text indicates a statistically significant correlation with a *p*-value less than 0.05.

**Table 5:** Multivariate logistic regression analysis for specific cut-off values of biochemical parameters.

	OR	*p*
**hs-CRP (mg/L)**	**47.225**	**0.001**
**sIL-2R (ng/mL)**	4.029	0.158
**ACE (ng/mL)**	4.445	0.201

For hs-CRP cut-off value: 1.8 mg/L; For sIL-2R cut-off value: 5.80 ng/mL; For ACE cut off value: 26.23 ng/mL. Bold text indicates a statistically significant correlation with a *p*-value < 0.05.
